# The hexosamine biosynthetic pathway alters the cytoskeleton to modulate cell proliferation and migration in aggressive prostate cancer

**DOI:** 10.1186/s12964-026-02756-9

**Published:** 2026-03-28

**Authors:** Rajina Shakya, Praveen Suraneni, Amit Rahi, Michael Y. Young, Emily Cousino, Christine B. Magdongon, Raju Gajjela, Basil B. Mattamana, Alexander Zaslavsky, Ganesh S Palapattu, Arun Sreekumar, Dileep Varma

**Affiliations:** 1 Department of Cell and Developmental Biology, Feinberg School of Medicine, Northwestern University, Chicago, IL 60611 USA; 2 Department of Urology, University of Michigan Medical School, Ann Harbor, MI 48108 USA; 3 Proteomics Core, Feinberg School of Medicine, Northwestern University, Chicago, IL 60611 USA; 4 Department of Molecular and Cellular Biology and Alkek Center for Molecular Discovery, Baylor College of Medicine, Houston, TX 77030 USA

**Keywords:** Hexosamine biosynthetic pathway, Metastatic prostate cancer, Cell proliferation, Cytoskeleton, Mitosis, Cell migration, Cell signaling

## Abstract

Castration-resistant prostate cancer (CRPC) progresses despite androgen deprivation therapy, as cancer cells adapt to grow without testosterone, becoming more aggressive and prone to metastasis. CRPC biology complicates the development of effective therapies, posing challenges for patient care. Recent gene-expression and metabolomics studies highlight the Hexosamine Biosynthetic Pathway (HBP) as a critical player, with key components like GNPNAT1 (Glucosamine-phosphate N-acetyltransferase 1) being downregulated in CRPC. GNPNAT1 knockdown in pre-clinical models has been shown to increase growth and metastasis in CRPC tumors, though the mechanisms remain unclear.

To investigate the cellular basis of these CRPC phenotypes, we generated a CRISPR-Cas9 knockout model of GNPNAT1 in 22Rv1 CRPC cells, analyzing its impact on transcriptomic and glycoproteomic profiles of cells. We find that HBP inhibition disrupts the cytoskeleton, altering mitotic progression and promoting uncontrolled growth. GNPNAT1 KO cells showed reduced levels of cytoskeletal filaments, such as actin and microtubules, leading to cell structure disorganization and chromosomal mis-segregation. GNPNAT1 inhibition also activated PI3K/AKT signaling, promoting cell proliferation, impaired cell adhesion by mis-localizing Eph Receptor B6, enhancing migration via the RhoA (Ras homolog family member A) pathway and promoting epithelial-to-mesenchymal transition. These findings suggest that HBP plays a critical role in regulating CRPC cell behavior, and targeting this pathway could provide a novel therapeutic approach.

**Supplementary Information:**

The online version contains supplementary material available at 10.1186/s12964-026-02756-9.

## Introduction

Prostate cancer (PCa) predominantly affects men over 50, particularly those with a family history of disease, who face a higher risk [1]. Most PCa initially respond well to standard androgen deprivation therapy which lowers levels of male hormones, specifically testosterone [2]. However, due to increased mutational load and genomic instability, prostate tumors develop resistance to this treatment over time, leading to castration-resistant prostate cancer (CRPC) [3]. In CRPC, cancer cells find ways to thrive and proliferate often signaling an advanced disease state with a higher likelihood of metastasis. These cancer cells retain the ability to survive even in the absence of circulating androgens making treatment more challenging [4]. Thus, a personalized and multidisciplinary approach that takes into account the unique characteristics of the CRPC is essential for managing complications and optimizing treatment outcomes.

The interplay between metabolic pathways and cytoskeletal function is critical in tumor progression and metastasis [5]. Cancer cells often undergo metabolic reprogramming to meet their increased energy and biosynthetic demands with enhanced lipid metabolism being a notable example [6, 7]. However, the mechanisms through which these metabolic pathways control PCa are unclear [8]. While it is known that metabolic shift influences the cytoskeleton by modulating actin dynamics, the role of microtubule cytoskeleton in promoting malignant properties, especially in PCa is largely unexplored [9]. For example, elevated levels of fatty acid synthase (FASN) in prostate cancer have been associated with changes in cytoskeletal structure that enhance cell motility and invasiveness [7]. Additionally, prostate cancer cells typically exhibit increased glucose metabolism, which supplies key metabolites like ATP and acetyl-CoA to promote cytoskeletal reorganization. Rho GTPases, which regulate actin cytoskeleton remodeling, are often upregulated in prostate cancer, promoting cell migration and facilitating metastasis [5, 7]. These findings suggest that targeting metabolic pathways that affect cytoskeletal dynamics may be a promising therapeutic strategy for advanced prostate cancer.

The hexosamine biosynthetic pathway (HBP) is a metabolic pathway that branches off from glycolysis and plays a crucial role in nutrient sensing and cellular regulation [10]. The HBP is sensitive to nutrient availability and cellular stress, making it a crucial player in cellular responses to changing metabolic conditions [11]. This pathway converts glucose to UDP-N-acetylglucosamine (UDP-GlcNAc), a key building block for glycosylation reactions. UDP-GlcNAc is involved in various cellular processes, including the modification of proteins through N/O-GlcNAcylation which regulates the function of many proteins, influencing processes such as signal transduction, transcription, and cell cycle progression. Dysregulation of the HBP has been implicated in cancer, affecting various aspects of tumor development and progression [10–12]. The pathway intersects with key cellular processes and signaling pathways, influencing cancer cells in several ways, including changes in glycosylation patterns, Insulin resistance, and metabolic rewiring [13]. In prostate cancer, the HB’s dysregulation contributes to altered cellular metabolism that enhances tumor growth [8]. The rate-limiting step of HBP involves the conversion of Gluocosamine-6-phosphate to N-acetylglucosamine-6-phosphate which is catalyzed by Glucosamine 6-phosphate N-acetyltransferase (GNPNAT1) [14]. An earlier study demonstrated that HBP pathway is downregulated in CRPC and knock down (KD) of GNPNAT1 in CRPC increased tumor growth and metastasis [8].

In this study, we demonstrate that the reduction in HBP alters both actin as well as the microtubule cytoskeleton and enhances AKT and MAPK levels, to regulate cell morphology, proliferation, and motility in different CRPC model systems.

## Materials and methods

### Cell lines and cell culture

The human normal prostate cell line RWPE1, human prostate cancer cell line DU145, and human CRPC cell lines 22Rv1 and LNCaP were purchased from ATCC and cultured in RPMI culture media with phenol red (Life Technologies) supplemented with 10% fetal bovine serum (Seradigm, VWR LifeScience), and 1% penicillin/streptomycin (Seradigm, VWR LifeScience). The LNCaP-abl *wt* and LNCaP-abl GNPNAT1 shRNA knockdown cell lines were kindly provided by our collaborator, Dr. Arun Sreekumar, and were grown in 10% charcoal stripped FBS without phenol red. The wild-type along with the GNPNAT1 knockout/knockdown cell lines were grown at 37 °C in a 5% CO_2_ humidified incubator.

### Chemical and reagents

Primary antibodies against GNPNAT1 and RND3 were purchased from ProteinTech whereas AKT, p-AKT, ERK, p-ERK, RhoA, RhoB, and RhoC were purchased from Cell Signaling Technology (Denver, CO, USA). Antibodies against β-actin and α-tubulin were obtained from Santa Cruz Biotechnology (Dallas, TX, USA). Antibodies against GAPDH was purchased from Millipore (Burlington, MA, USA). EphB6, E-Cadherin (ECAD), N-Cadherin (NCAD), Paxillin, Integrin β1, Integrin β4, Androgen Receptor, MMP9, and MMP12 from Abclonal (Woburn, MA, USA). MK2206 was purchased from AdooQ Bioscience (Irvine, CA) and UDP-GlcNAc from Millipore sigma (Burlington, MA).

### CRISPR/Cas9-mediated knockout

The gRNAs targeting the human GNPNAT1 gene in 22Rv1, RWPE1, and DU145 cells were designed using the CRISPR-based Gene Knockout Kit v2 from Synthego Corporation. The sequences for the multiguide RNAs were: U*A*U*UUGAACAAAAACAGAAG, U*A*C*UUACUCAUAAAUUGUUC, U*U*C*AAAACUAGGUUUUUUUA. To prepare the cells, 22Rv1 CRPC cells were seeded and grown to the required confluency in an incubator at 37 °C with 5% CO₂. The GNPNAT1 gRNAs were combined with spCas9 2NLS protein at room temperature (RT) for 20 min to form Cas9 ribonucleoprotein (RNP) complexes. These RNP complexes were introduced into the 22Rv1 cells via electroporation using the Neon NxT Electroporation System (ThermoFisher Inc.) following the manufacturer’s instructions from Synthego. As a positive control, a non-targeting gRNA provided by Synthego was used. Post-electroporation, the cells were cultured for 24–48 h before being sorted into single cells in 96-well plates containing RPMI medium supplemented with 20% FBS. The growth of single-cell colonies was monitored over 4–6 weeks, allowing the cells to expand into colonies. These colonies were progressively scaled up to 24-well and then 6-well plates. The resulting cells were split into two groups: one for cryopreservation and the other for validation using western blot analysis to confirm the presence of homozygous knockout clones.

### Immunofluorescence microscopy

Cells were fixed on coverslips using either 100% ice-cold methanol or 3.6% paraformaldehyde. Initially, the coverslips were rinsed three times with 1x PBS. For methanol fixation, cells were pre-treated with ice-cold methanol for 1 min, followed by incubation at − 20 °C for 5 min. Alternatively, for paraformaldehyde fixation, cells were first permeabilized with 0.5% Triton X-100 for 5 min, then fixed with 3.6% paraformaldehyde for 20 min at RT. Blocking was carried out using 0.1% BSA in PBS for 1 h at RT. The cells were then immunostained with a primary antibody overnight at 4 °C, followed by three washes with 1x PBS for 10 min each. Next, the cells were incubated with Alexa Fluor-488/647 or Rhodamine Red-X secondary antibodies (Thermo Fisher Scientific) for 1 h at 37 °C, as needed. After two additional PBS washes, the nuclei or chromosomes were counterstained with DAPI (1:10,000 dilution in 1x PBS) for 10 min at RT. Finally, the coverslips were mounted onto glass slides using ProLong Gold Antifade reagent (Thermo Fisher Scientific) as the mounting medium. The slides were stored at 4 °C until imaging.

### Image acquisition and processing

For fixed-cell imaging, three-dimensional image stacks were captured using a Nikon Eclipse TiE inverted microscope fitted with a Yokogawa CSU-X1 spinning disc, an Andor iXon Ultra888 EMCCD camera, and either a 60x or 100 × 1.4 NA Plan-Apochromatic DIC oil immersion objective (Nikon). Imaging was conducted at RT with Z-stacks taken at 0.2–0.3 μm intervals, managed through the NIS-Elements software (Nikon). The captured images were processed in NIS-Elements and displayed as maximum-intensity projections of the relevant Z-stacks. Image intensities were quantified using ImageJ Fiji, and the resulting data were plotted into graphs using Origin 2018 software for further analysis.

### Cell spreading assay

Live-cell imaging of the cell spreading assay in control and GNPNAT1 KO 22Rv1 cells was performed using 35-mm glass-bottom dishes (MatTek Corporation) coated manually with 10 µg/ml fibronectin before cell culture. Imaging was conducted in an incubated chamber (Tokai Hit Co., Ltd) at 37 °C with 5% CO₂, using FluoroBrite DMEM live imaging media (Life Technologies) supplemented with 2.5% FBS. Images were captured every 15 min for up to 6 h, depending on whether the samples were control or GNPNAT1 KO 22Rv1 cells.

### Western Blotting

The cells were collected, washed with PBS, and lysed using RIPA buffer (Sigma-Aldrich, #R0278) supplemented with Halt Protease Inhibitor Cocktail (Thermo Scientific, #87786), followed by incubation on ice for 20 min. The lysates were centrifuged at 14,000 rpm for 5 min at 4 °C, and the supernatant was collected. Protein concentrations were measured using the Coomassie protein assay, and samples were mixed with Laemmli Sample Buffer, then boiled at 100 °C for 10 min. Proteins were separated on a 12–15% SDS-PAGE gel and transferred onto PVDF membranes (Cytiva Amersham Hybond, #10600023). For western blotting, the PVDF membranes were blocked with 5% non-fat dry milk in 1x TBS containing 0.1% Tween-20 (TBST) for 1 h at RT with shaking, followed by three washes with 1x TBST for 10 min each. Primary and secondary antibodies were diluted in 1x TBST with 5% BSA. The membranes were incubated with the appropriate primary antibodies for 1 h at RT with shaking, washed three times with 1x TBST, and then incubated with goat anti-rabbit (Azure Biosystems, #AC2114) or goat anti-mouse HRP-conjugated secondary antibodies (Azure Biosystems, #AC2115) at a 1:2000 dilution for 1 h with shaking at RT. Protein detection was performed using SuperSignal West Pico PLUS Chemiluminescent Substrate (Thermo Fisher Scientific, #34580).

### Cell proliferation assay

The cell proliferation in 22Rv1, RWPE1, and DU145 cells was measured using the Cell Proliferation Reagent WST-1 kit from Millipore Sigma (Burlington, MA). In brief, 10⁴ cells were seeded into quadruplicate wells of a 96-well plate and incubated at 37 °C with 5% CO₂ in a humidified atmosphere for the specific period. The WST-1 reagent was diluted five-fold in the culture media, and 200 µL of the solution was added to each well. The plate was then incubated for 2 additional hours, after which the optical density (OD) was measured at 450 nm using a Promega microplate reader.

### RNA sequencing analysis

RNA was extracted from WT and GNPNAT1 KO cells using the Direct-zol™ RNA MiniPrep kit (Zymo Research, #R2050). RNA quality was assessed at the NUSeq Core facility at Northwestern University. Library preparation, sequencing, and data analysis were carried out using the Standard RNA-seq services provided by GENEWIZ, Inc. The resulting data were visualized as a Volcano plot, which was generated using the R programming software.

### Glycoproteomics analysis

Immunoprecipitation of N-glycosylated proteins: To a 1000 µl Cerebellar lysate (1 µg/µl), 20 µg/ml biotinylated Concanavalin (Con-A) was added, and the reaction mixture was incubated overnight at 4 °C rotating end over end. NeutrAvidin agarose beads (29204, ThermoFisher, Scientific-25 µl) were used to recover Con-A bound proteins at 4 °C for 1 h. The mixture was spun at 2500 g for 2 min to collect the beads. The beads were washed with PBS 3 times followed by elution of N-glycosylated proteins in 2.5X SDS buffer by boiling the samples at 95 °C for 10 min. After immunoprecipitation, N-glycosylated proteins were sent to Northwestern University Proteomics Core facility where Samples were precipitated overnight using eight volumes of acetone and one volume of TCA. The resulting protein pellet was resuspended and vortexed in 100 µL of 8 M urea and 0.4 M ammonium hydrogen bicarbonate (AmBic) solution. Reduction was performed by adding 4 µL of 100 mM dithiothreitol (DTT) and incubating for 30 min at 55 °C. Alkylation was carried out in the dark using 18 mM iodoacetamide for 30 min at RT. The urea concentration was then reduced to 1.8 M by adding four volumes of water. Digestion occurred overnight at 37 °C using MS-grade trypsin (Promega, Madison, WI) at an enzyme-to-substrate ratio of 1:50. To halt the digestion, 10% formic acid was added to achieve a final concentration of 0.5%. The peptides were desalted using Pierce C-18 spin columns and eluted with 80% ACN/0.1% FA, then dried in a vacuum concentrator. The dried peptides were resuspended in 30 µL of 5% ACN/0.1% FA for LC-MS analysis with a Dionex UltiMate 3000 Rapid Separation nano-LC paired with a Q Exactive HF (QE) Quadrupole Orbitrap mass spectrometer (Thermo Fisher Scientific Inc., San Jose, CA, USA). Identified peptides and proteins were visualized using Scaffold software (version 5.0, Proteome Software Inc., Portland, OR). The data were searched against a particular database using the MaxQuant application. For statistical analysis, a t-test was performed with a significance threshold of *p* < 0.05 and a fold change (FC) greater than 2 to identify significantly up- and down-regulated proteins, which were visualized using a volcano plot.

### Proteome array analysis

Proteome array analysis was conducted for Human Phospho-Kinase proteome array according to the manufacturer’s instruction. In brief, cells were harvested and lysed with ice cold lysis buffer 17 provided with the Human Phospho-RTK proteome array kit (Bio-techne, Minneapolis, USA) for 30 min on ice. The samples were then centrifuged at 14,000 rpm for 10 min, supernatant was collected, and quantification of protein was carried out using Coomassie protein assay. The arrays were then blocked for 1 h by Array buffer 1 included in the kit, which serves as a blocking buffer. Then, they were incubated with 600 µg protein samples overnight at 4 °C. The unbounded proteins on array were removed by washing three times with washing buffer. Arrays were incubated with anti-phospho-tyrosine-HRP detection antibody for 2 h at RT on a rocking platform shaker followed by three times washing with wash buffer. After the final wash, the excess wash buffer was allowed to drain from the array and Chemi Reagent Mix was added evenly onto each membrane. Multiple exposures were carried out to visualize the protein spot and the average intensity was calculated using ImageJ software program (Bethesda, Maryland, USA). After subtracting the averaged background signal, the fold change was obtained by comparing GNPNAT1 KO cells with control 22Rv1 cells.

### Wound healing assay

The wound healing assay was conducted using the Ibidi culture-insert 2-well system (Lochhamer Schlag, Germany). Initially, cells were harvested and seeded at a final concentration of 3 × 10⁵ cells/ml, then incubated at 37 °C with 5% CO₂ for 24 h. After incubation, the culture insert was carefully removed, and pre-warmed, cell-free medium (37 °C) was gently added by pipetting. The cells were then allowed to migrate, and their movement was monitored using phase contrast microscopy. Imaging began with the acquisition of single frames at various time points (0, 6, 24, 48, and 72 h) depending on the rate of migration.

### Cell migration and invasion assay

Initially, the inner surface of the Transwell insert (BD Falcon, Franklin Lakes, USA) was coated with collagen (1 mg/ml) for the cell migration assay and with Matrigel (0.5 mg/ml) for the cell invasion assay. Following coating, 100 µl of cell suspension (5 × 10⁵ cells/ml) in serum-free media was added to the inner chamber of the Transwell and incubated at 37 °C with 5% CO₂ for 48 h. After the 48-hour incubation, migrated and invaded cells were treated with the WST cell proliferation assay kit, and absorbance was measured at 440 nm using a Promega microplate reader.

### In vivo Tumor Xenograft and UDP treatment

All animal procedures were conducted in accordance with the Institutional Animal Care and Use Committee (IACUC)] guidelines at the University of Michigan. Male castrated Nod-SCID Gamma mice (6–8 weeks old) from Charles River Laboratories were utilized for the subcutaneous xenograft model. 22Rv1 GNPNAT1-knocked down (KD) cells (5 × 10^5) resuspended in a 1:1 ratio of PBS and Matrigel was injected subcutaneously between the shoulder blades. Upon the development of palpable tumors, mice were randomized into two groups for treatment. The experimental group received 60 mM UDP dissolved in PBS via oral gavage every other day, while the control group received an equivalent volume of PBS vehicle alone. Tumor volumes were monitored with digital calipers, and volumes were determined using the standard formula:

$$\mathrm{Volume}\;=\;(\mathrm W^2\ast\mathrm L)/2$$

Statistical significance of the growth curves was analyzed using a two-way ANOVA to evaluate the interaction between treatment type and time.

### Statistical analysis

Data are presented as the mean standard deviation. Single comparisons were performed using the student’s t-test. Graphs and data plots were designed using Origin 2018 software. Statistical significance relative to the control group was indicated by *p* < 0.05 (*) and *p* < 0.01 (**).

## Results

### HBP inhibition modulates cell proliferation, adhesion, and migration properties

**Fig. 1:**
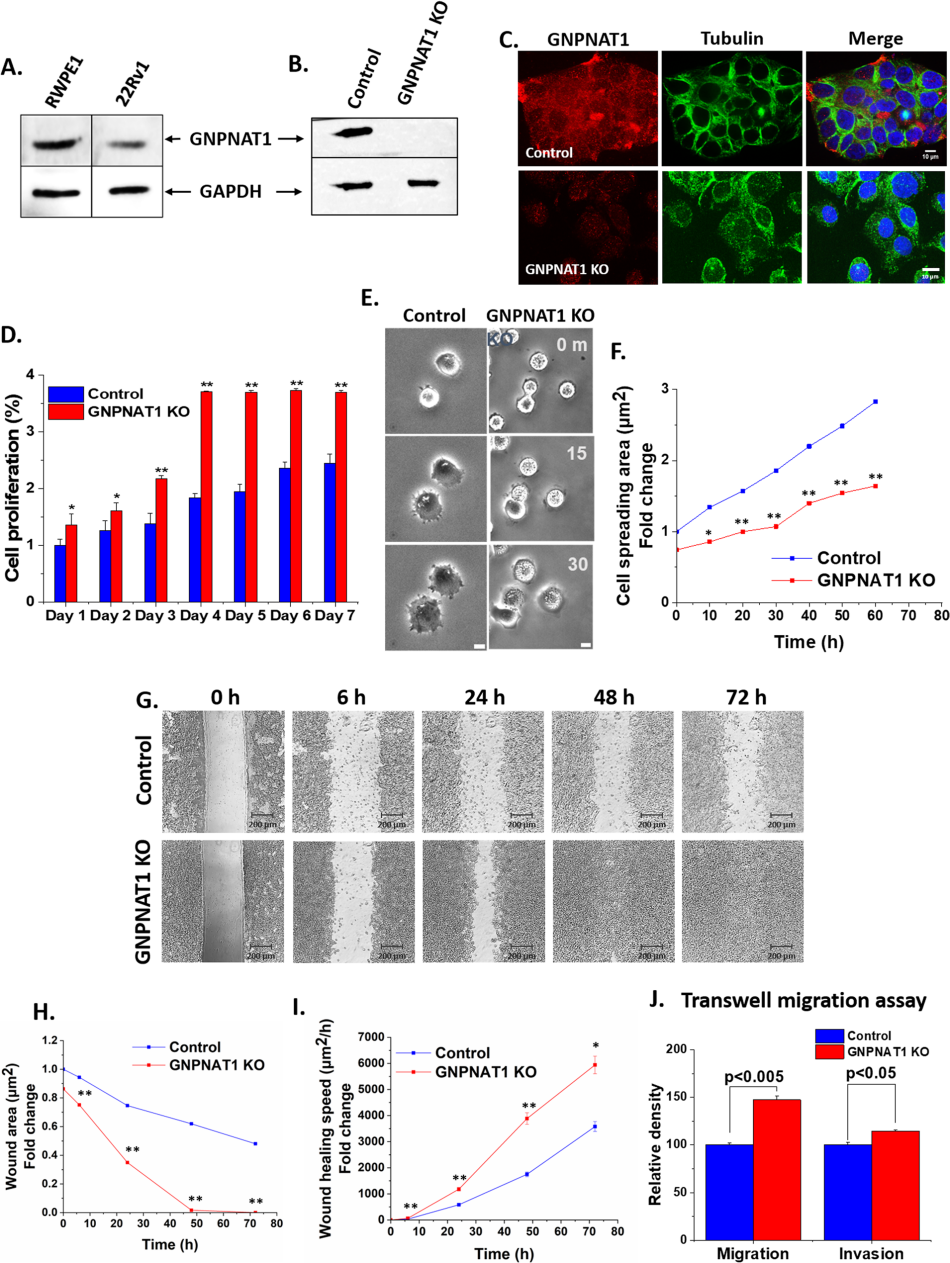
HBP depletion alters various genes, metabolites, glycoproteins as well as their associated pathways and processes. **A** Western blot showing the downregulation of GNPNAT1 in CRPC (22Rv1) cells as compared to normal (RWPE1) cells. **B**, **C** Confirmation of CRISPR CAS9-mediated knockout of GNPNAT1 in 22Rv1 cells by (**B**) western blotting and (**C**) immunofluorescence staining. Scale bar, 10 μm. **D** Cell proliferation assay of control and GNPNAT1 KO 22Rv1 cells at days 1–7. Statistical significance compared to the control group is shown by *p < 0.05 and **p < 0.01. **E**-**F** Microscopic images (**E**) and quantification of cell spreading (**F**) performed on fibronectin-coated glass surface. Scale bars, 25 μm. **G**-**I** Microscopic images (**G**) and graph showing wound area (**H**) and wound healing speed (**I**) observed by the wound healing assay. Scale bars, 200 μm. **J** Transwell migration and invasion assay of control and GNPNAT1 KO 22Rv1 cells

Previous studies have implicated alterations in the hexosamine biosynthetic pathway (HBP) as key contributors to the progression of castration-resistant prostate cancer (CRPC). Consistent with these findings, we observed a reduction in GNPNAT1 expression in 22Rv1 CRPC cells compared to the normal prostate cell line, RWPE1 (Fig. 1A). Downregulation of the HBP pathway in CRPC cells has been linked to enhanced cell proliferation and invasion [8]. However, the physiological basis of these cancer-associated phenotypes is unclear. To address this, we generated a CRISPR-mediated GNPNAT1 knockout (KO) in 22Rv1 cells, which was confirmed via western blotting and immunofluorescence (Fig. 1B-C). Consistent with the previously published report [8], GNPNAT1 KO cells exhibited a significant increase in cell proliferation over time as compared to the control 22Rv1 cells (Fig. 1D). Using the same approach, we knocked out GNPNAT1 in DU145, a human prostate cancer cell line and RWPE1, a normal prostate cell line (Supplementary Figure S1A-B), where we also observed a similar increase in cell proliferation in GNPNAT1 KO cells over time (Supplementary Figure S1D-E).

Morphological analysis revealed that GNPNAT1 KO 22Rv1 cells appeared more rounded or elongated and occupied less surface area compared to controls (Supplementary Figure S1C). To investigate the effects of GNPNAT1 KO on cell adhesion to the substratum, we performed a cell adhesion assay in which we monitored the spreading of GNPNAT1 KO and control 22Rv1 cells on fibronectin-coated glass-bottom dishes. Time-lapse imaging of cell morphology indicated that the 22Rv1 KO cells spread at a much slower rate as compared to the controls cells, suggesting that the cell adhesion to the substratum had been impaired in the KO cells (Fig. 1E-F). Further, we observed that GNPNAT1 KO cells demonstrated enhanced cell migration properties in 22Rv1 cells (Fig. 1G-I) and RWPE1 cells (Supplementary Figure S1F-H). There was both a substantial decrease in wound area and an increase in wound healing speed in GNPNAT1 KO cells as compared to control cells (Fig. 1H-I). Since the wound healing assay only represents cohort cell migration, we performed trans-well cell invasion/migration assay to confirm an effect on individual cell migration. Based on this assay, we observed that cells migrate and invade more in GNPNAT1 KO cells in comparison to control cells in 22Rv1 cells (Fig. 1J).

### HBP inhibition disrupts the cytoskeletal integrity and induces defective mitosis

**Fig. 2:**
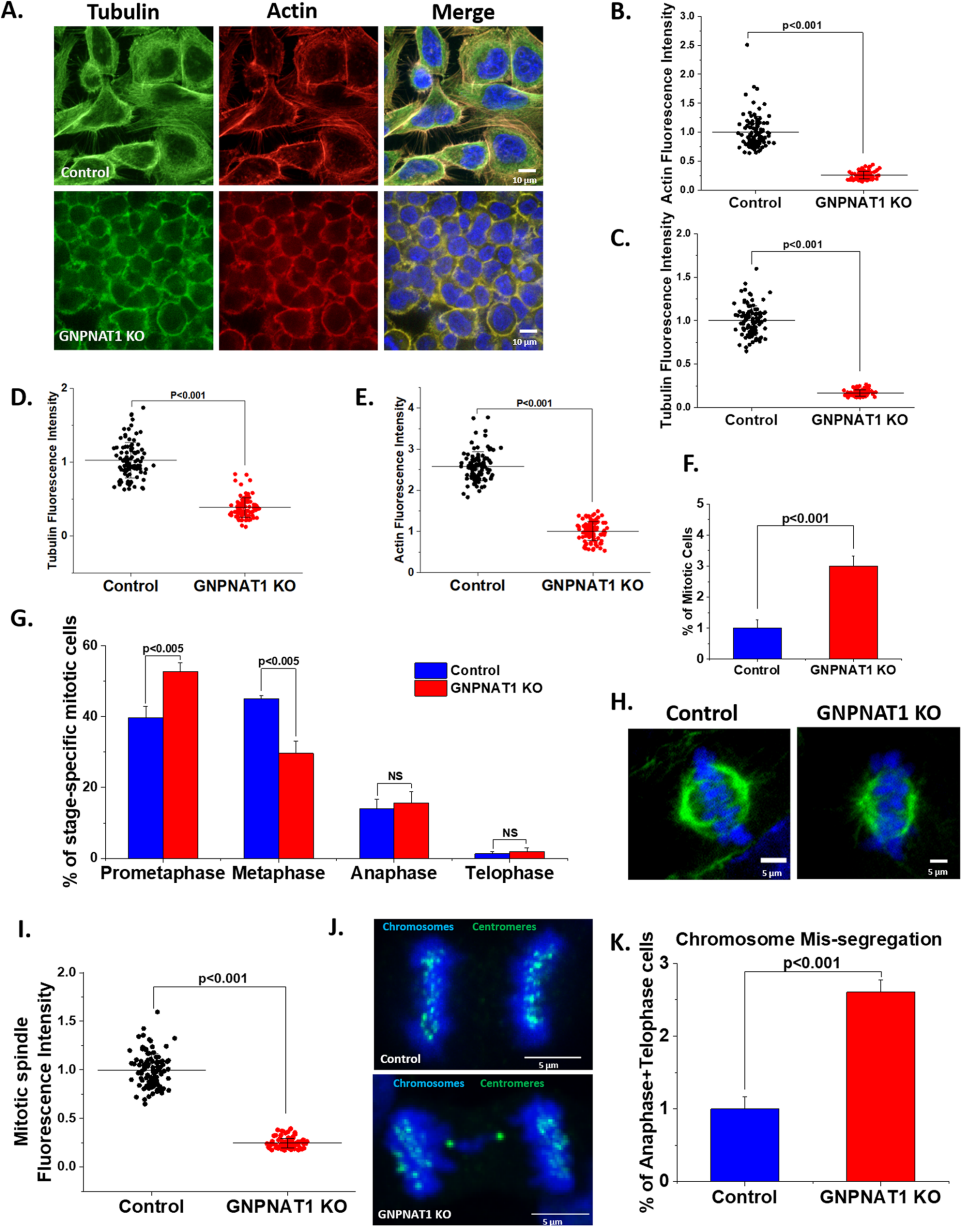
HBP depletion disrupts both actin as well as the microtubule cytoskeleton and increases mitotic frequency. **A** Immunostaining of the actin and microtubule cytoskeleton in control and GNPNAT1 KO 22Rv1 cells. Scale bars, 10 μm. **B**-**C** Quantification of intensity of individual actin (**B**) and microtubule (**C**) filaments after immunostaining. **D**-**E** Quantification of intensity of individual microtubule (**D**) and actin (**E**) filaments after immunostaining in control and GNPNAT1 knockdown LNCaP-abl cells. **F** Mitotic cell count of control and GNPNAT1 KO cells, represented as the Mitotic Index (number of mitotic cells/100 total cells). **G** Stage specific mitotic cell counts of control and GNPNAT1 KO cells. **H** Immunofluorescence staining of mitotic spindles in control and GNPNAT1 KO cells. Scale bar, 5 μm. **I** Quantification of total microtubule intensity from F. **J** Control and GNPNAT1 KO cells were immuno-stained with a centromere marker and the chromosomes marked using DAPI. Scale bar, 5 μm. **K** Quantification of the frequency of chromosome mis-segregation events from J

We noticed that GNPNAT1 KO 22Rv1 cells displayed profound disruptions in actin and microtubule organization. Immunostaining revealed significant downregulation of both cytoskeletal components in KO cells, which were localized primarily to the cell periphery rather than the cytosol (Fig. 2A-C). A similar reduction in the intensity of actin and microtubules was observed in GNPNAT1 KO RWPE1 (Supplementary Figure S2A-C), DU145 cells (Supplementary Figure S2D-F) and in LNCaP-abl cells after GNPNAT1 knockdown (Fig. 2D-E; Supplementary Figure S2G-H), indicating that downregulation of the HBP significantly disrupts the cytoskeleton.

As HBP inhibition has been shown to promote cell proliferation, we sought to investigate the downstream alterations in cellular properties that facilitated the alteration in mitotic characteristics of CRPC cells. The total frequency of proliferating mitotic cells increased significantly in GNPNAT1 KO cells (Fig. 2F). This change was accompanied by an enrichment of prometaphase cells, and a reduction in metaphase cells (Fig. 2G). Further, the mitotic spindle structure was markedly disorganized, with a threefold reduction in spindle microtubule intensity and a significant decrease in spindle size (Fig. 2H-I; Supplementary Figure S2I). Chromosomal mis-segregation, a hallmark of aneuploidy, was observed to be threefold higher in GNPNAT1 KO cells as compared to controls (Fig. 2J-K).

To further validate the impact of HBP-related metabolites on tumor progression in a more physiological context, we performed a subcutaneous xenograft study. Consistent with our in vitro findings, treatment with UDP-GlcNAc significantly suppressed tumor growth over a 22 day period compared to vehicle control (PBS) (Supplementary Figure S3; *p* = 0.00047, Two-way ANOVA). These results demonstrate that the HBP-associated metabolite UDP-GlcNAc exerts a potent inhibitory effect on tumor cell expansion in vivo, reinforcing the pathway’s role as a critical regulator of aggressive prostate cancer progression.

### Transcriptomic and Glycoproteomic Analyses of GNPNAT1 KO cells

**Fig. 3:**
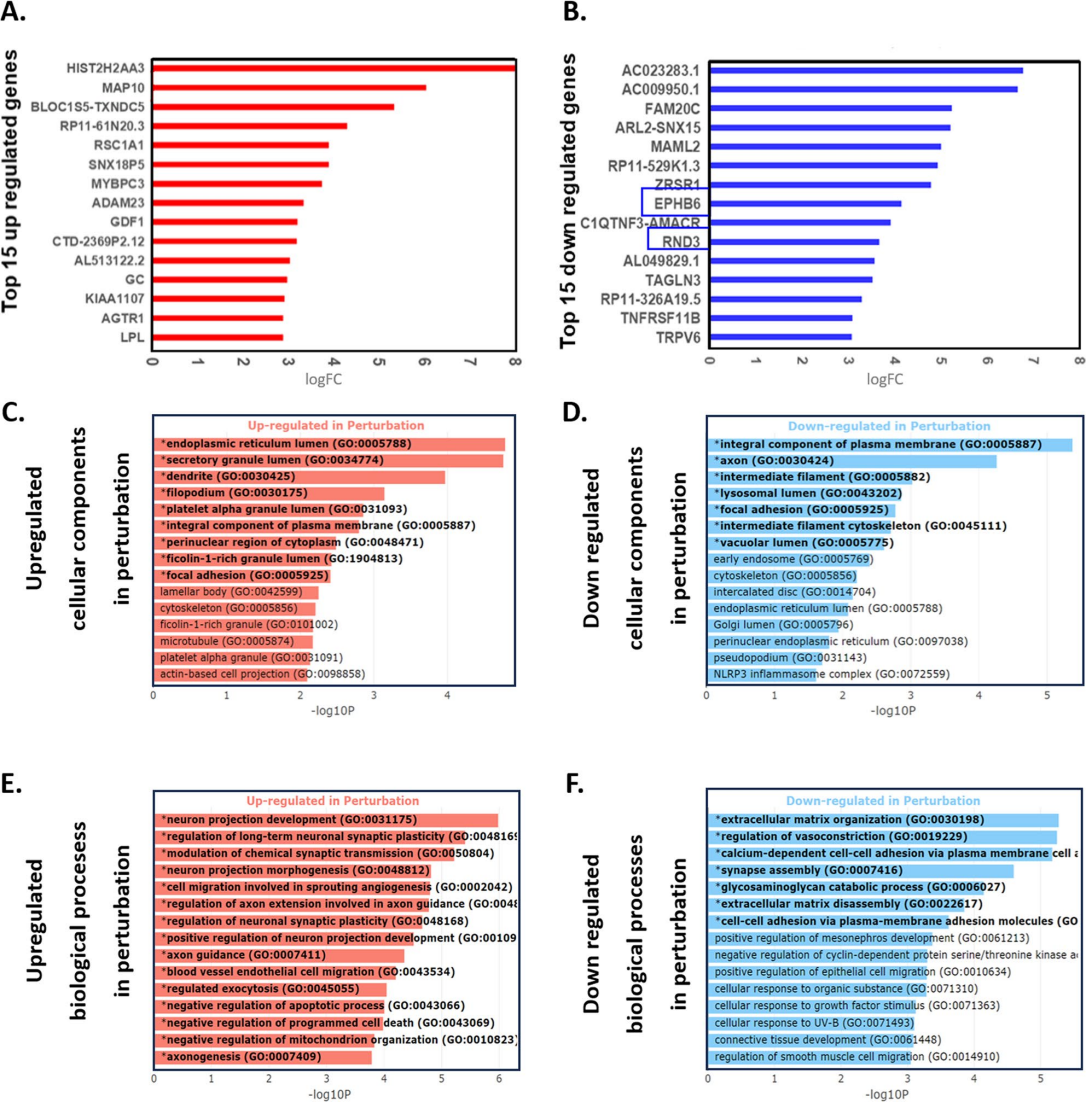
HBP depletion affects gene regulation. **A-B** Representation of the top 15 down-regulated (**A**) and up-regulated genes (**B**) via differential gene expression analysis of GNPNAT1 depleted 22Rv1 cells. **C-D** Gene ontology analysis of downregulated (**C**) and upregulated (**D**) cellular components as well as upregulated (**E**) and downregulated (**F**) biological processes

Since there is only limited data available on how GNPNAT1 inhibition affects various cellular phenotypes, we carried out transcriptomics analysis to better comprehend the cellular characteristics of GNPNAT1-inhibited cells. We used differential gene expression analysis in order to identify the genes that are specifically associated with the depletion of GNPNAT1. 994 genes were found to be differentially expressed in the transcriptome of GNPNAT1 KO cells, implicating widespread transcriptional reprogramming (Supplementary Figure S4A). Key DEGs included Eph receptor B6 (EphB6) and Rho family GTPase 3 (RND3), which were significantly downregulated, along with alterations in other cell signaling and cytoskeletal genes. (Fig. 3A-B). EphB6 and RND3 are key regulators of cell signaling pathways involved in cell adhesion, migration, and cytoskeletal dynamics. EphB6 is part of the Eph/ephrin signaling pathway, while RND3 modulates Rho GTPase signaling. These pathways are critical for controlling cancer cell motility and invasiveness, thus being central to our experimental focus.

Gene Ontology Analysis of biological processes suggests that several gene groups closely associated with cell adhesion and migration were severely dysregulated (Fig. 3C-F). GNPNAT1 KO 22Rv1 cells showed markedly altered pathways, including upregulated cell migration and downregulated extracellular matrix organization and disassembly, cell-cell adhesion, and glycosaminoglycan metabolism (GAGs). Further, glycoproteomic profiling revealed substantial alterations in cellular glycosylation patterns in GNPNAT1 KO 22Rv1 cells. Our analysis revealed that glycosylation of multiple cellular components closely linked to the cytoskeleton, signaling, cell adhesion and migration were altered (Supplementary Figure S4B-C). These findings underscore the broad impact of GNPNAT1 loss on processes and pathways controlling diverse cellular properties.

### GNPNAT1 KO enhances cell migration via EMT and the modulation of signaling pathways

**Fig. 4:**
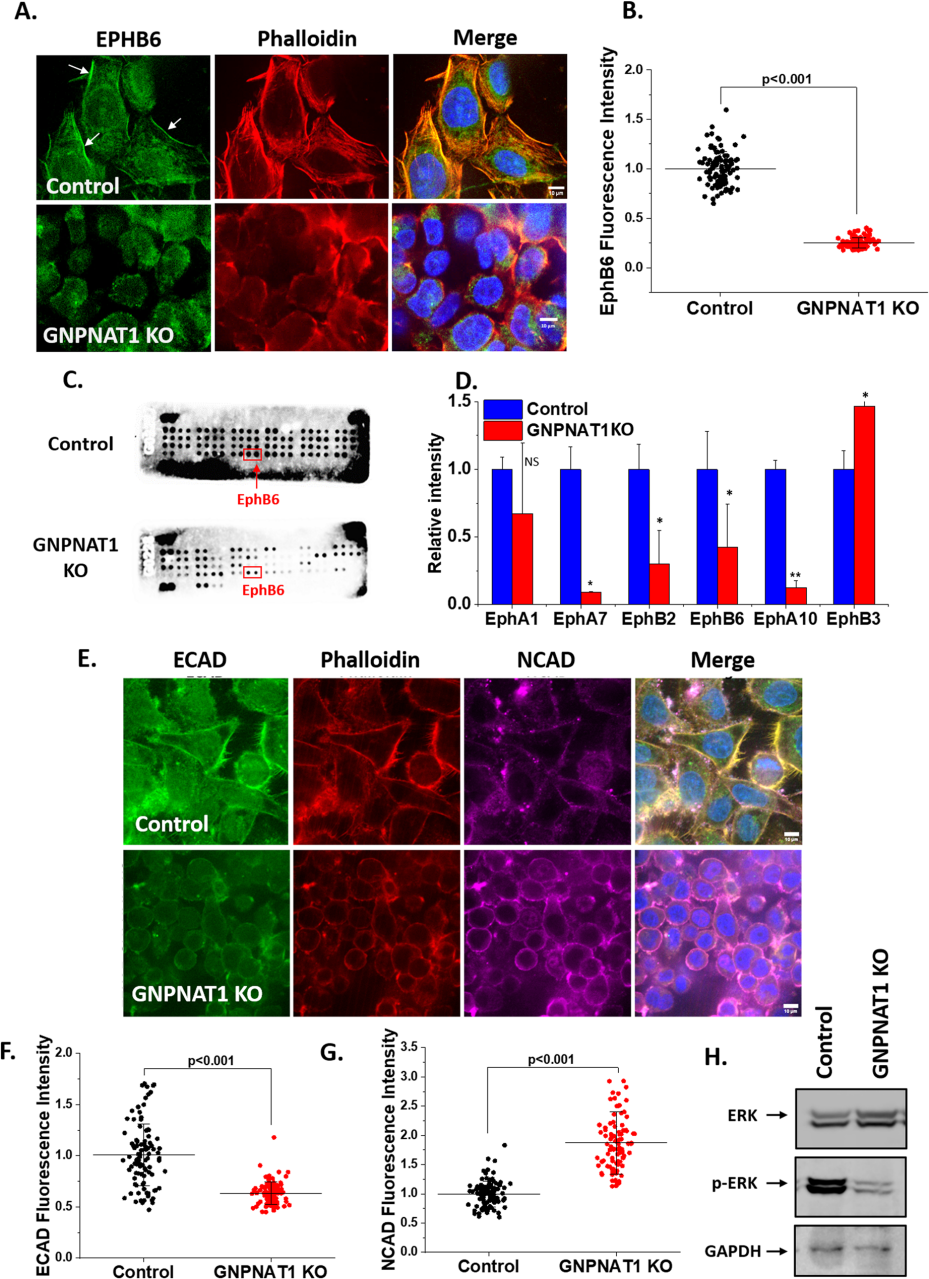
HBP depletion reduces EphB6 ERK signaling and promotes Epithelial-to-Mesenchymal Transition (EMT). **A** EphB6 immunofluorescence staining with DAPI in control and GNPNAT1 KO 22Rv1 cells. Arrows indicate brighter EphB6 staining at the cell periphery. Scale bars, 10 μm. **B** Quantification of EphB6 levels at the cell periphery from A. **C**-**D** Quantification of protein expression level of multiple Ephrins using Human RTK proteome array. Statistical significance compared to the control group is shown by *p < 0.05 and **p < 0.01. **E** ECAD, NCAD and Phalloidin-actin immunofluorescence staining with DAPI in control and GNPNAT1 KO 22Rv1 cells. Scale bars, 10 μm. **F**-**G** Quantification of ECAD and NCAD levels at the cell periphery from E. **H** Expression levels of total and phosphorylated form of ERK in control and GNPNAT1 KO 22Rv1 cells as indicated

Ephrin and Eph receptors are overexpressed in a variety of human tumors, as shown by multiple studies [15, 16]. On the other hand, tumor development has been associated with both their up- and down-regulation, and both ephrin ligands and Eph receptors have the ability to either promote or inhibit the growth of tumors [17, 18]. We confirmed that EphB6 is indeed downregulated in GNPNAT1 KD cells as suggested by our RNA sequencing data (Fig. 4A-B). We believe that this is a key contributing factor in the reduction of cell adhesion properties. Further, we observed that several other Eph receptors, such as EphA1, EphA7, EphB2, and EphA10 were also downregulated in addition to EphB6 after GNPNAT1 KO, while EphB3 was upregulated as demonstrated by the RTK proteome array (Fig. 4C-D), which is consistent with the transcriptomics results. EphB6 is a kinase-defective Eph receptor known to function as a tumor suppressor, modulating cell adhesion, migration, and invasion. Its loss is linked to enhanced metastatic behavior [19, 20]. Members of the EphA subgroup such as EphA1 & EphA7 are involved in cell–matrix adhesion and migration, with roles in epithelial–mesenchymal transition (EMT) in other cancers [21]. Like EphB6, EphB2 contributes to contact-dependent cell repulsion, helping maintain epithelial barriers and inhibit migration. Though less well-studied, EphA10 has been identified as a target for tumor therapy and may impact cell-cell interactions [20]. Interestingly, though found to be upregulated in our array, some EphB receptors have dual roles where their upregulation can promote re-adhesion or alter EMT dynamics [22].

Our transcriptomics results also led us to insinuate that epithelial-to-mesenchymal transition (EMT) is crucial to the observed cellular phenotype as we had identified notable changes in the extracellular matrix (ECM) assembly and disassembly pathways. Immunofluorescence analyses illustrate that the ECAD levels were lower while NCAD levels were elevated in GNPNAT1 KO 22Rv1 cells (Fig. 4E-G). A similar pattern of reduced ECAD and increased NCAD expression was also observed in GNPNAT1-depleted RWPE1 (Supplementary Figure S5A-D) and DU145 cell lines (Supplementary Figure S5E-H), indicating a consistent shift towards a mesenchymal-like phenotype across different prostate cell models. The results suggest that EMT phenotype in CRPC cells is indeed influenced by GNPNAT1 downregulation, as evidenced by the immunofluorescence data for E- and N-cadherin. We also find that the levels of phosphorylated ERK was reduced in GNPNAT1 inhibited cells (Fig. 4H). Based on these results, we speculate that downregulation of EphB6 activated the MAPK/ERK signaling pathway to alter cell surface cadherins and drive the loss of adhesion, which in turn might facilitate cell migration (see model in Fig. 8).

**Fig. 5:**
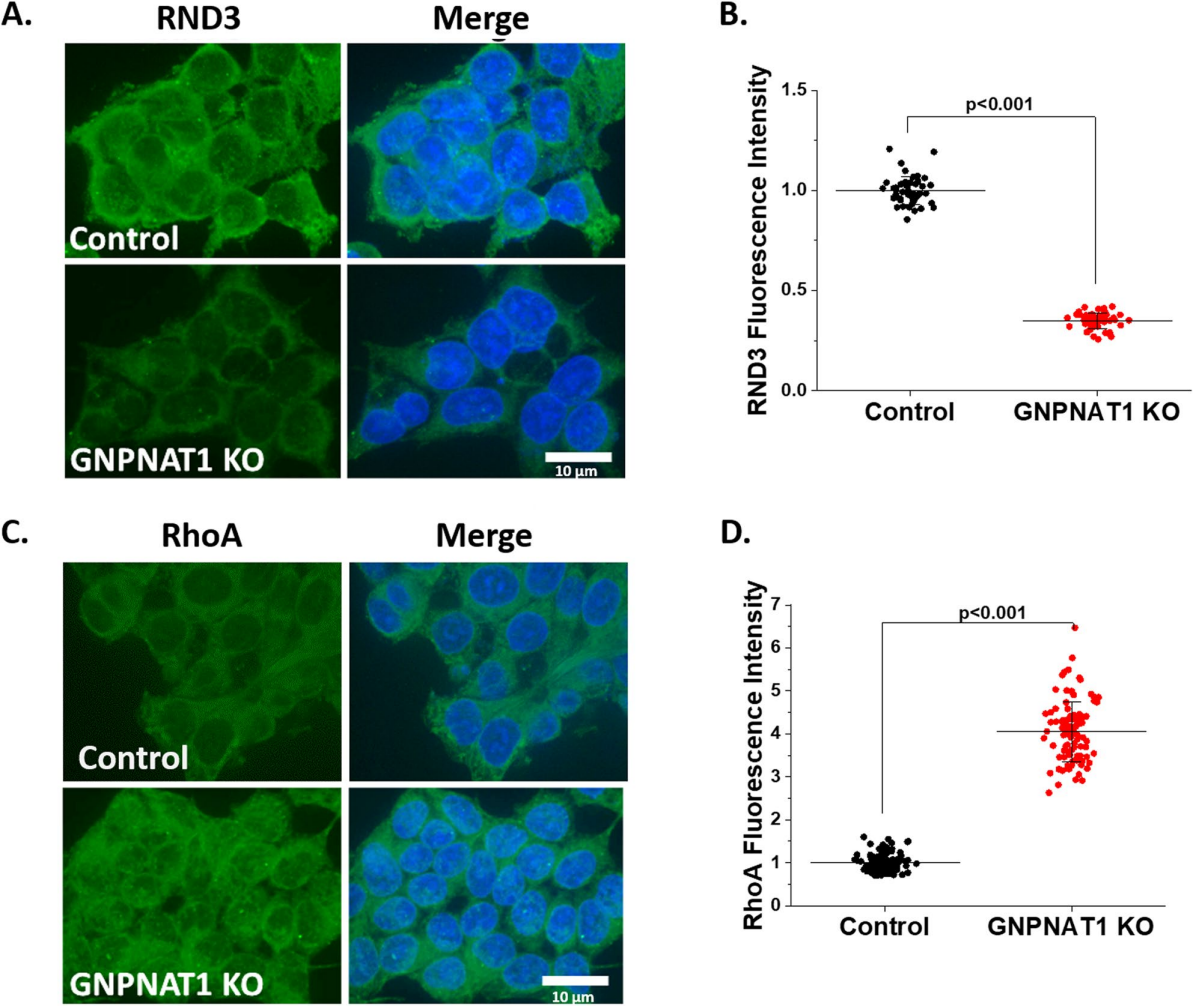
HBP depletion alters RND3/RhoA signaling. **A** RND3 immunofluorescence staining with DAPI in control and GNPNAT1 KO 22Rv1 cells. Scale bars, 10 μm. **B** Quantification of RND3 cytoplasmic levels from A. **C** RhoA immunofluorescence staining with DAPI in control and GNPNAT1 KO 22Rv1 cells. Scale bars, 10 μm. **D** Quantification of RhoA cytoplasmic levels from C

As initially suggested by our RNA seq results, our immunofluorescence staining experiments confirmed that the expression of the RND3 is reduced in GNPNAT1-inhibited cells. These experiments also showed that RhoA levels increase in the same condition (Fig. 5A-D). It is known that downregulation of RND3 leads to increased RhoA activity which enhances cell migration, invasion, and other mesenchymal behaviors through epithelial to mesenchymal transition [23–26]. However, other Rho members such as RhoB and RhoC were found to be reduced after GNPNAT1 inhibition (Supplementary Figure S6A-D).

**Fig. 6:**
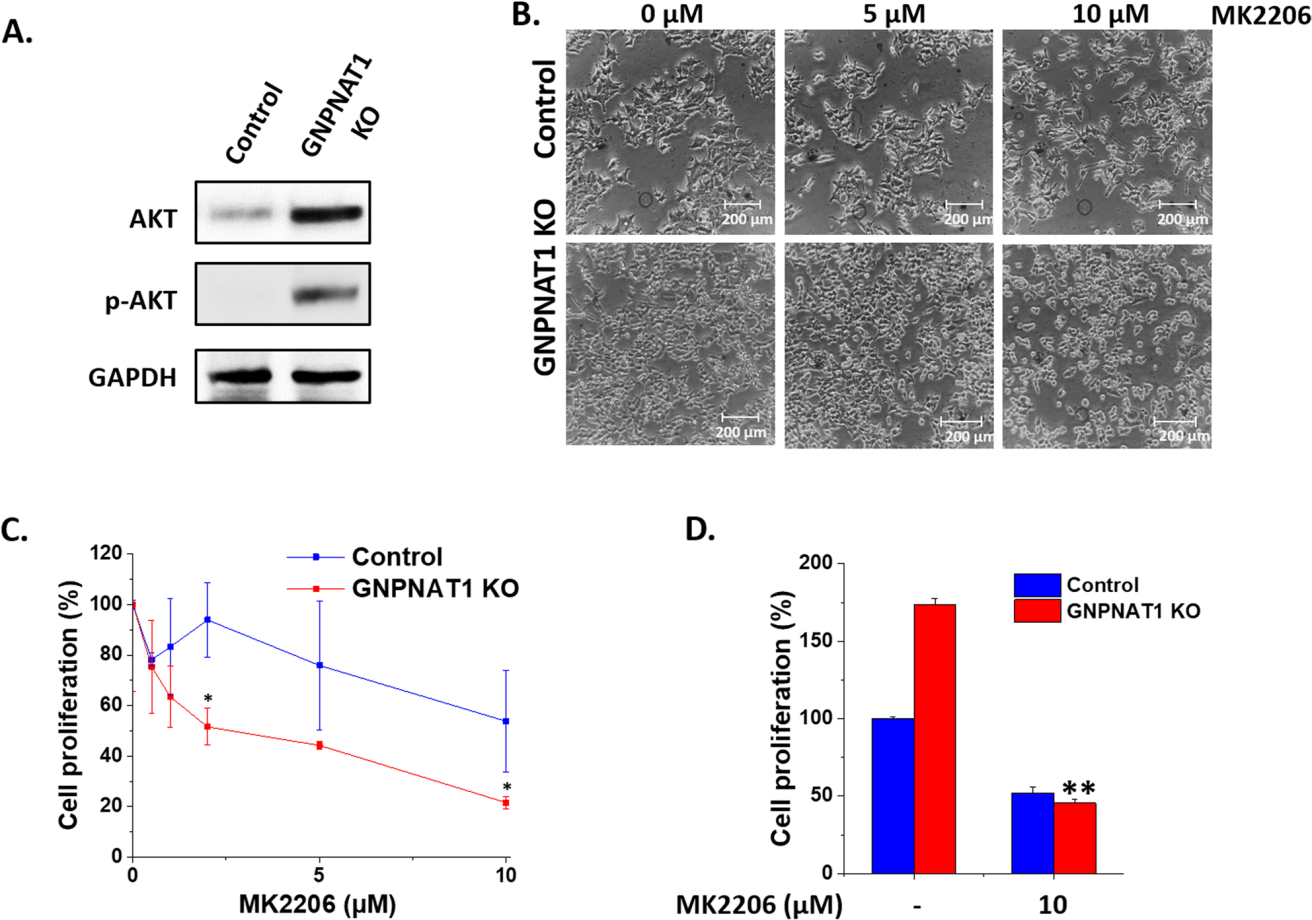
HBP depletion promotes cell proliferation by altering AKT-associated signaling pathways. **A-C** Control and GNPNAT1 KO 22Rv1 cell lysate samples were Western blot-analyzed for the levels of AKT and its phosphorylated form p-AKT (**A**). **B**-**C **Microscopic images (**B**) and cell proliferation assay (**C**) after treating control and GNPNAT1 KO with the different concentrations of the AKT inhibitor, MK2206 for 48 h. Scale bars, 200 μm. **D** Cell proliferation assay after treatment with 10 µM MK2206 for 48 h, highlighting the difference between control and GNPNAT1 KO

Previous studies have indicated that the PI3K/AKT pathway is activated in GNPNAT1 inhibited cells [8]. Our results confirmed this finding as AKT was upregulated in both its total and phosphorylated forms in GNPNAT1 KO 22Rv1 (Fig. 6A), as well as in RWPE1 and DU145 cells (Supplementary Figure S7). Consistent with that, we found that treatment with the AKT inhibitor MK2206 significantly reduced cell proliferation in GNPNAT1 KO 22Rv1 cells (Fig. 6B-D).

**Fig. 7:**
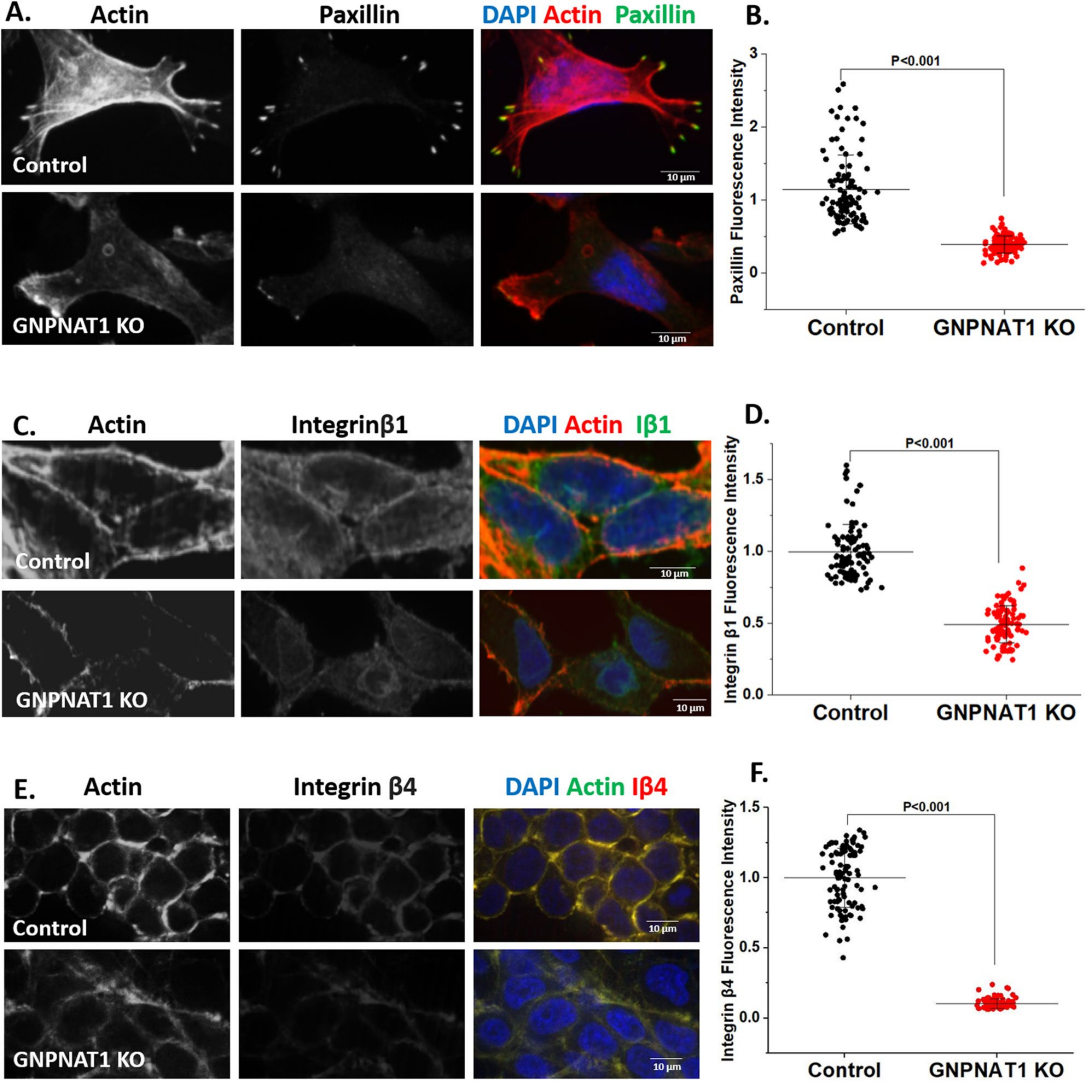
HBP depletion impedes cell adhesion by suppressing cellular levels of paxillin and integrin. **A** Paxillin immunofluorescence staining in control and GNPNAT1 knockdown LNCaP-abl cells. Scale bars, 10 μm. **B** Quantification of paxillin levels at focal adhesions of cells from A. **C** Integrin β1 immunofluorescence staining in control and GNPNAT1 knockdown LNCaP-abl cells. Scale bars, 10 μm. **D** Quantification of cell surface Integrin β1 levels from C. **E** Integrin β4 immunofluorescence staining in control and GNPNAT1 KO 22Rv1 cells. Scale bars, 10 μm. **F** Quantification of cell surface Integrin β4 levels from E

To further investigate the mechanism underlying the enhanced cell proliferation and migration, we hypothesized that these phenotypes may be associated with altered cell–matrix interactions, including changes in cell adhesion molecules and extracellular matrix (ECM) remodeling [27]. We thus examined the expression of key cell adhesion- and invasion-related markers, including paxillin, integrin β1, integrin β4, and matrix metalloproteinases (MMPs). Our results showed that the expression of paxillin, and integrin β1 was markedly reduced in LNCaP-abl GNPNAT1 knockdown cells compared with control cells (Fig. 7A-D). Similar results were observed for integrin β4 in GNPNAT1 KO 22Rv1 cells (Fig. 7E-F). In contrast, the expression levels of MMP9 and MMP12 were significantly increased in LNCaP-abl GNPNAT1 knockdown cells relative to controls (Supplementary Figure S8A-D), suggesting enhanced ECM remodeling and invasive potential.

## Discussion

The hexosamine biosynthetic pathway (HBP) is a key metabolic pathway where glucose is transported into cells, phosphorylated to glucose-6-phosphate, and isomerized to fructose-6-phosphate. Amidation of fructose-6-phosphate with glutamine produces glucosamine-6-phosphate, which is catalyzed by GNPNAT1 to form N-acetylglucosamine-6-phosphate (GlcNAc-6-P). This compound is subsequently converted into N-acetylglucosamine-1-phosphate (GlcNAc-1-P) and ultimately UDP-N-acetylglucosamine (UDP-GlcNAc). UDP-GlcNAc plays a central role in glycosylation, protein modification, signal transduction, and protein stability [11, 28, 29].

Previous work linking metabolic pathways and prostate cancer had combined gene-expression, mouse in vivo studies and metabolomics analysis on benign and prostate cancer tissues [8, 29]. Compilation of gene-expression and metabolomic data showed overall enrichment of five pathways including riboflavin metabolism, biotin metabolism, amino sugar metabolism (HBP), valine, leucine, and isoleucine biosynthesis, and cysteine metabolism. Among various pathways studied, the HBP was found to be the most highly dysregulated metabolic pathway. This analysis was based on data from the KEGG (Kyoto Encyclopedia of Genes and Genomes) database, which maps biological systems. The finding highlights the importance of the HBP in regulating key biological functions due to its extensive involvement in various cellular interactions [8]. Several studies have observed the upregulation of gene-expression profiles of HBP genes such as GNPNAT1, UAP1 (UDP-N-Acetylglucosamine Pyrophosphorylase 1), and UDP-GluNAc in localized prostate cancer tissue [14, 30–32]. However, the expression profiles of these genes were significantly reduced in metastatic or CRPC tumor tissue. Further, GNPNAT1 knockdown leads to increased cell proliferation, invasion, tumor growth and metastasis of 22Rv1 and LNCaP-abl CRPC cell lines [8].

The exact mechanism of elevated cell proliferation and metastasis of HBP-inhibited cells however remain unknown. In this study, CRISPR-Cas9-mediated GNPNAT1 knockout in human CRPC cells revealed profound changes in cellular morphology, enhanced proliferation, and reduced adhesion, as evidenced by cell spreading assays. Wound-healing and invasion assays demonstrated that GNPNAT1-deficient cells exhibited heightened migratory and invasive potential, underscoring the critical role of HBP in these processes. One plausible explanation for the increased proliferation observed in GNPNAT1 knockout cells is the elevated number of mitotic cells. This could be attributed to accelerated mitotic entry or delayed progression, leading to mitotic accumulation. The smaller mitotic spindles and reduced spindle microtubule intensity observed in GNPNAT1 knockout cells may expedite chromosome segregation, yet compromise segregation accuracy, as evidenced by increased chromosome mis-segregation and aneuploidy. Aneuploidy, characterized by abnormal chromosome numbers, is typically associated with cellular dysfunction [33]. However, in certain contexts, it can paradoxically enhance proliferation via compensatory mechanisms such as altered gene expression, activation of signaling pathways, or modulation of cell cycle checkpoints [34–36].

Cytoskeletal dynamics, governed by actin and microtubules, are critical for mitosis, proliferation, and migration [37–39]. Similar to our observations in 22Rv1 cells, loss of GNPNAT1 in the CRPC model LNCaP-abl was associated with reduced microtubule and actin levels. This suggests that the effect of GNPNAT1 on cytoskeletal organization is consistent across different CRPC models. These findings also support a role for GNPNAT1 in maintaining cytoskeletal integrity in advanced prostate cancer. In GNPNAT1 knockout cells, disrupted actin and microtubule organization likely contributed to impaired adhesion and altered motility. These cytoskeletal changes disrupt intracellular structural networks, affecting cell shape, motility, and division, as well as key downstream signaling pathways. Eph receptors and their ephrin ligands play crucial roles in cell communication, migration, and adhesion during development and tissue homeostasis [17]. EphB6, as a member of the Eph receptor family, can activate intracellular signaling pathways, including the MAPK/ERK pathway [21–24]. HBP-inhibited cells were found to have reduced EphB6 levels. The loss of EphB6 could either lead to the upregulation or activation of other signaling pathways or target its downstream signaling that enhance cell migration while negatively impacting adhesion [18]. This downregulation may suppress ERK phosphorylation, impairing adhesion while enhancing migration. Further, HBP dysregulation has also been implicated in activation of the PI3K/AKT signaling pathway, a critical regulator of cell survival, proliferation, and cytoskeletal organization [8, 40]. While our study confirmed the increase in AKT activity in HBP-inhibited CRPC cells, it is not clear if this is directly related to the reduction in EphB6 signaling.

GNPNAT1 deficiency was linked to epithelial-to-mesenchymal transition (EMT), a process associated with metastasis. EMT involves a shift from epithelial to mesenchymal states, characterized by decreased epithelial markers (e.g., E-cadherin) and increased mesenchymal markers (e.g., N-cadherin), resulting in enhanced motility and invasiveness [41, 42]. Elevated RhoA activity and reduced RND3 levels, a RhoA antagonist, in GNPNAT1 knockout cells likely contributed to EMT, facilitating enhanced migration and proliferation.

Our observations regarding the HBP-dependent regulation of cytoskeletal dynamics and mitotic fidelity are further supported by the functional validation of the UDP metabolite in a physiological context. Given that GNPNAT1 depletion leads to an aggressive proliferative phenotype in vitro, we sought to determine if restoring metabolic homeostasis via exogenous metabolite administration could revert this effect in vivo. Treatment with the HBP-associated metabolite UDP significantly attenuated subcutaneous tumor growth in a xenograft model, compared to vehicle-treated controls (Supplementary Figure S3). These data suggest that the physiological concentrations of HBP metabolites, such as UDP and UDP, serve as a critical checkpoint for the morphological and signaling transformations that drive CRPC progression. By suppressing tumor growth in vivo, UDP counteracted the proliferative advantage gained through HBP deficiency, likely by stabilizing the intracellular structural networks and Rho-dependent signaling pathways identified as downstream effectors of the pathway in our cellular models.

In addition to cytoskeletal alterations, GNPNAT1 loss also affected key regulators of cell–matrix adhesion and extracellular matrix remodeling. Reduced expression of paxillin, integrin β1, and integrin β4 after the loss of GNPNAT1 function suggest impaired focal adhesion formation and weakened cell–ECM interactions [27, 43]. These changes likely contribute to the enhanced migratory phenotype observed in these cells. Concurrently, the upregulation of MMP9 and MMP14 indicate increased extracellular matrix degradation, possibly to in turn facilitate cell invasion [44, 45]. Together, these findings suggest that GNPNAT1 deficiency promotes a pro-migratory and invasive phenotype through coordinated disruption of adhesion signaling and extracellular matrix remodeling.

Although 22Rv1 cells are not derived from therapy-resistant tumors, their partial resistance and AR-variant expression make them a relevant model to interrogate mechanisms of chemotherapy tolerance. Our findings suggest that loss of GNPNAT1 may sensitize CRPC cells to taxane-based therapy (Supplementary Figure S9), warranting future investigation in resistant models.

Future studies should investigate whether GNPNAT1 depletion affects response to androgen deprivation therapy. The activation of AKT signaling, metabolic reprogramming, and epithelial-to-mesenchymal transition observed in GNPNAT1 KO cells are consistent with adaptive mechanisms employed by castration-resistant prostate cancer. Additionally, as the hexosamine biosynthetic pathway regulates O-GlcNAcylation-mediated androgen receptor signaling, examining GNPNAT1’s role under androgen-depleted conditions would provide valuable mechanistic insights. Such studies could be performed using LNCaP or VCaP cells cultured in charcoal-stripped serum to model androgen deprivation in vitro, complemented by castration models in vivo to assess the role of GNPNAT1 in treatment resistance.

**Fig. 8:**
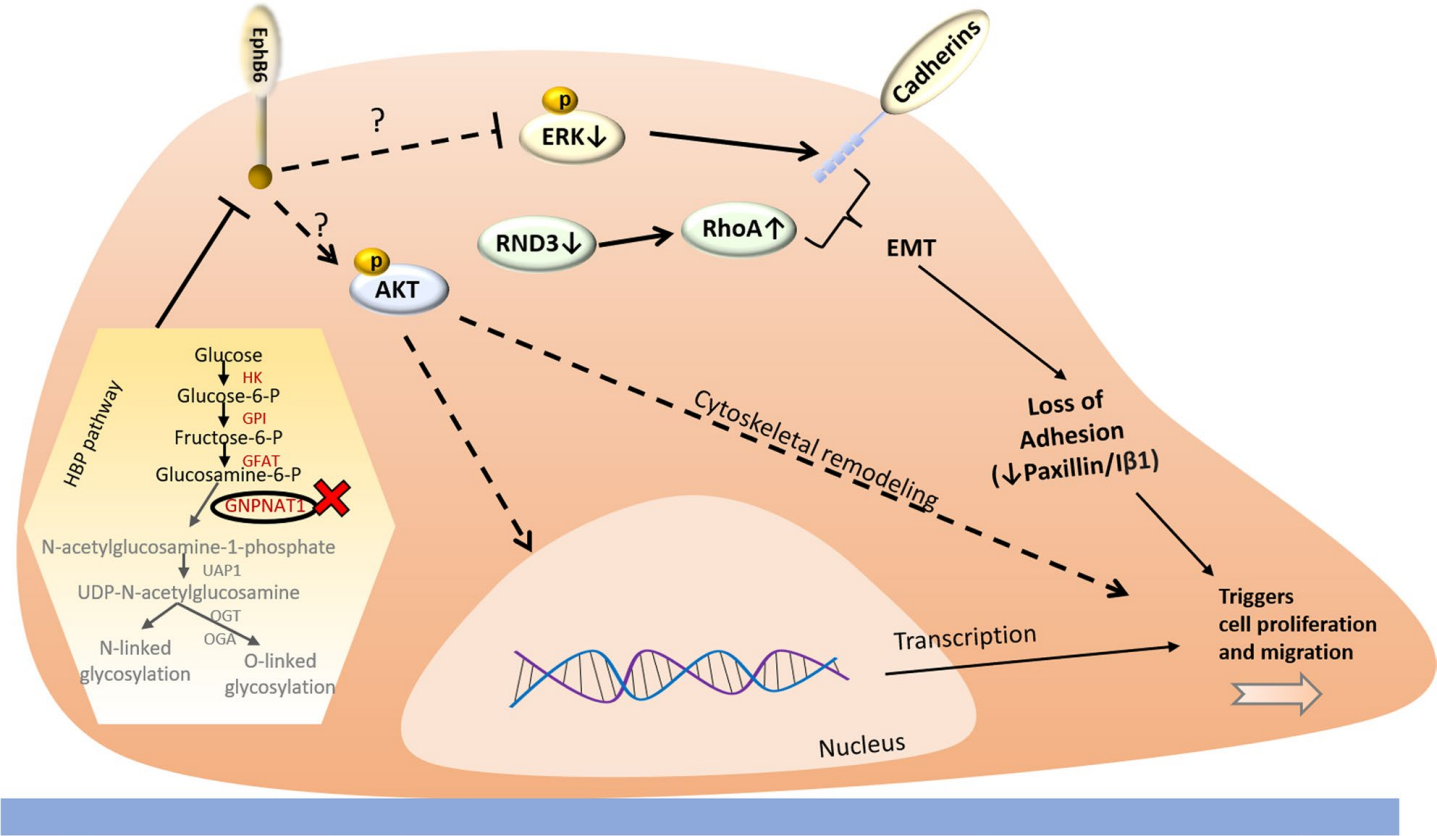
Schematic representation of the possible mechanism by which HBP controls cell proliferation and migration. Our observations suggest the HBP KO leads to the downregulation of EphB6 and the ERK pathway and activation of the AKT pathway thus contributing to the loss of cell adhesion and promoting cell proliferation as well as cell migration

Overall, our findings establish GNPNAT1 and the HBP as critical regulators of cytoskeletal organization, mitosis, cell adhesion, and migration in CRPC. These effects appear to be mediated through transcriptional reprogramming, altered glycosylation, EMT, and the activation of signaling pathways including PI3K/AKT, ERK/MAPK and RhoA (Fig. 8). These findings underscore the role of GNPNAT1 in maintaining cellular homeostasis and its dysregulation in promoting proliferation and metastasis [8, 14]. Future studies investigating the links between HBP and these pathways in greater detail may lead to the development of targeted therapies to mitigate CRPC progression.

## Supplementary Information

Supplementary Material 1. Supplementary Figure S1. HBP depletion affects the cell proliferation of RWPE1 and DU145 cell lines. (A-B) Confirmation of CRISPR CAS9-mediated knockout of GNPNAT1 in RWPE1 and DU145 cells by Western blotting. (C) Confocal microscopic images of control and GNPNAT1 KO 22Rv1 cells, showing cell morphology. Scale bars, 25 µm. (D-E) Cell proliferation assay of control and GNPNAT1 KO RWPE1 (D) and DU145 (E) cells at day 1-7. (F-H) Microscopic images (F) as well as graphs showing wound area (G) and wound healing speed (H) observed from the wound healing assay in F. Scale bars, 200 µm. Supplementary Figure S2. HBP depletion disrupts cytoskeleton in the RWPE1, DU145, and LNCaP-abl cell lines. (A) Immunostaining of the actin and microtubule cytoskeleton in control and GNPNAT1 KO RWPE1 cells. Scale bars, 10 µm. (B-C) Quantification of intensity of individual actin (B) and microtubule (C) filaments after immunostaining of RWPE1 cells. (D) Immunostaining of the actin and microtubule cytoskeleton in control and GNPNAT1 KO DU145 cells. Scale bars, 10 µm. (E-F) Quantification of intensity of individual actin (E) and microtubule (F) filaments after immunostaining of DU145 cells. (G-H) Immunostaining of the microtubule (G) and actin (H) cytoskeleton in control and GNPNAT1 knockdown LNCaP-abl cells. Scale bars, 10 µm. (I) Quantification of mitotic spindle area in control and GNPNAT1 KO 22Rv1 cells. Supplementary Figure S3: UDP Treatment inhibits subcutaneous tumor growth in vivo. Mice were implanted subcutaneously with tumor cells and monitored for volume growth over a 30-day period. Treatment with UDP (n=5) (open squares) resulted in a significant reduction in tumor volume compared to the PBS (n=4)-treated control group (black circles). Data are presented as mean tumor volume (W2*L)/2. Statistical significance was determined by two way ANOVA for the effect of treatment over time (p = 0.00047). Supplementary Figure S4. HBP inhibition affects various genes, metabolites, and glycoproteins associated with cell proliferation, adhesion and migration. (A) Volcano plot showing 994 genes being differentially expressed. (B-C) Total proteomic analysis of control and GNPNAT1 KO 22Rv1 cells showing the pathways and processes altered, including molecular function/interactions (C), and KEGG (Kyoto Encyclopedia of Genes and Genomes) pathway components (D). Supplementary Figure S5: HBP depletion promotes EMT in RWPE1 and DU145 cells. Immunofluorescence staining (A and C) and the intensity quantification (B and D) of ECAD and NCAD, respectively in control and GNPNAT1 KO RWPE1 cells. Scale bars, 10 µm. Immunofluorescence staining (E and G) and the intensity quantification (F and H) of ECAD and NCAD, respectively, in control and GNPNAT1 KO DU145 cells. Scale bars, 10 µm. Supplementary Figure S6: HBP depletion suppresses RhoB and RhoC levels. (A-D) Immunofluorescence staining (A and C) and the intensity quantification (B and D) of RhoB, and RhoC, respectively. Scale bars, 10 µm. Supplementary Figure S7: HBP depletion alters AKT signaling pathways. A Western blot analyzed for the levels of AKT and its phosphorylated form p-AKT in control and GNPNAT1 KO RWPE1 as well as DU145 cell lines, as indicated. Supplementary Figure S8: HBP depletion activates matrix metalloproteinase (MMP9 & MMP14). (A) MMP9 immunofluorescence staining in control and GNPNAT1 knockdown LNCaP-abl cells. Scale bars, 10 µm. (B) Quantification of cellular MMP9 levels from A. (C) MMP14 immunofluorescence staining in control and GNPNAT1 knockdown LNCaP-abl cells. Scale bars, 10 µm. (D) Quantification of cellular MMP14 levels from C. Supplementary Figure S9. Effectiveness of paclitaxel in control and GNPNAT1 KO 22Rv1cells. Cell proliferation assay of control and GNPNAT1 KO 22Rv1 cells at 24, 48, and 72 hrs after 0, 10, 50, 100, and 500 nM paclitaxel treatment, as indicated.

Supplementary Material 2.

## Data Availability

No datasets were generated or analysed during the current study.
