# Supplementary figures and images for: The hexosamine biosynthetic pathway alters the cytoskeleton to modulate cell proliferation and migration in aggressive prostate cancer
Source: Cell Commun Signal. 2026 Mar 28;24:311. doi: 10.1186/s12964-026-02756-9 (PMC13217896; doi:10.1186/s12964-026-02756-9)

# Figure 1A.

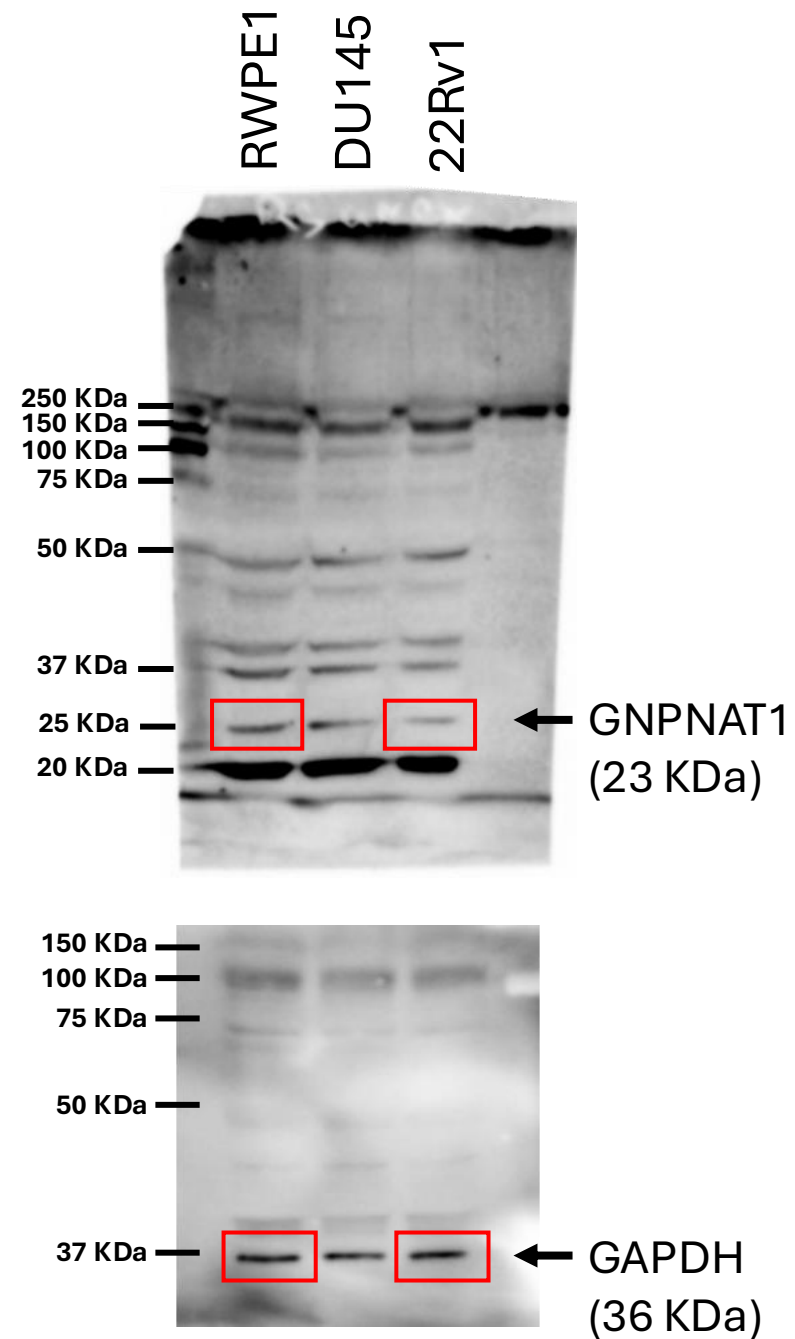

# Figure 1B.

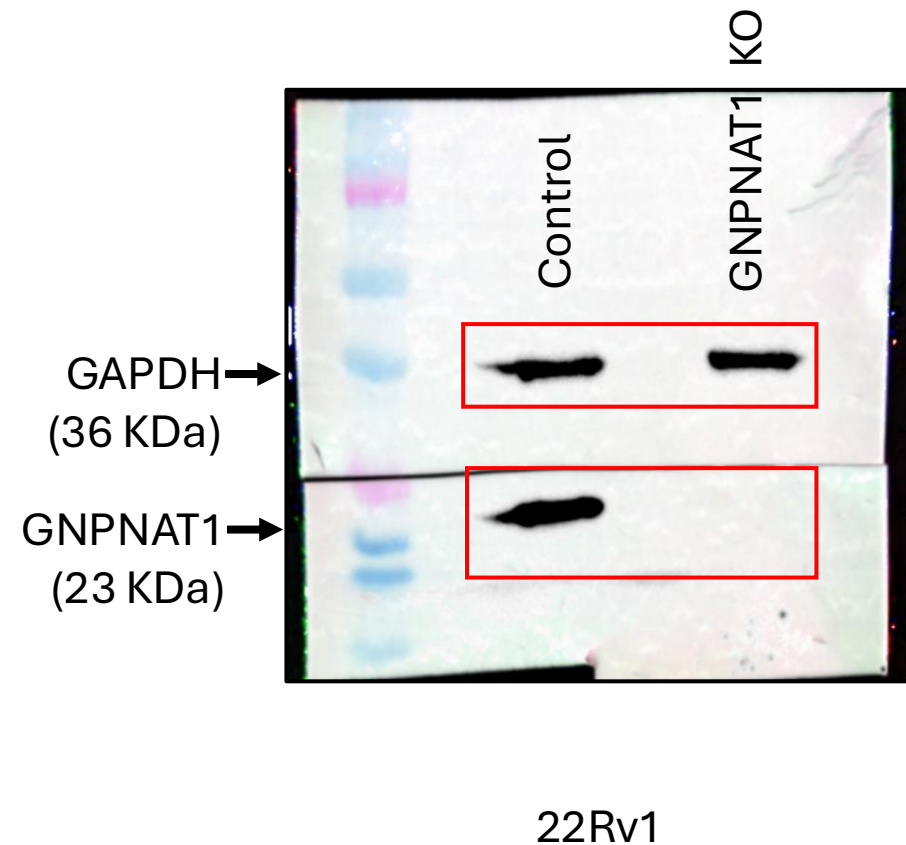

# Figure 4H.

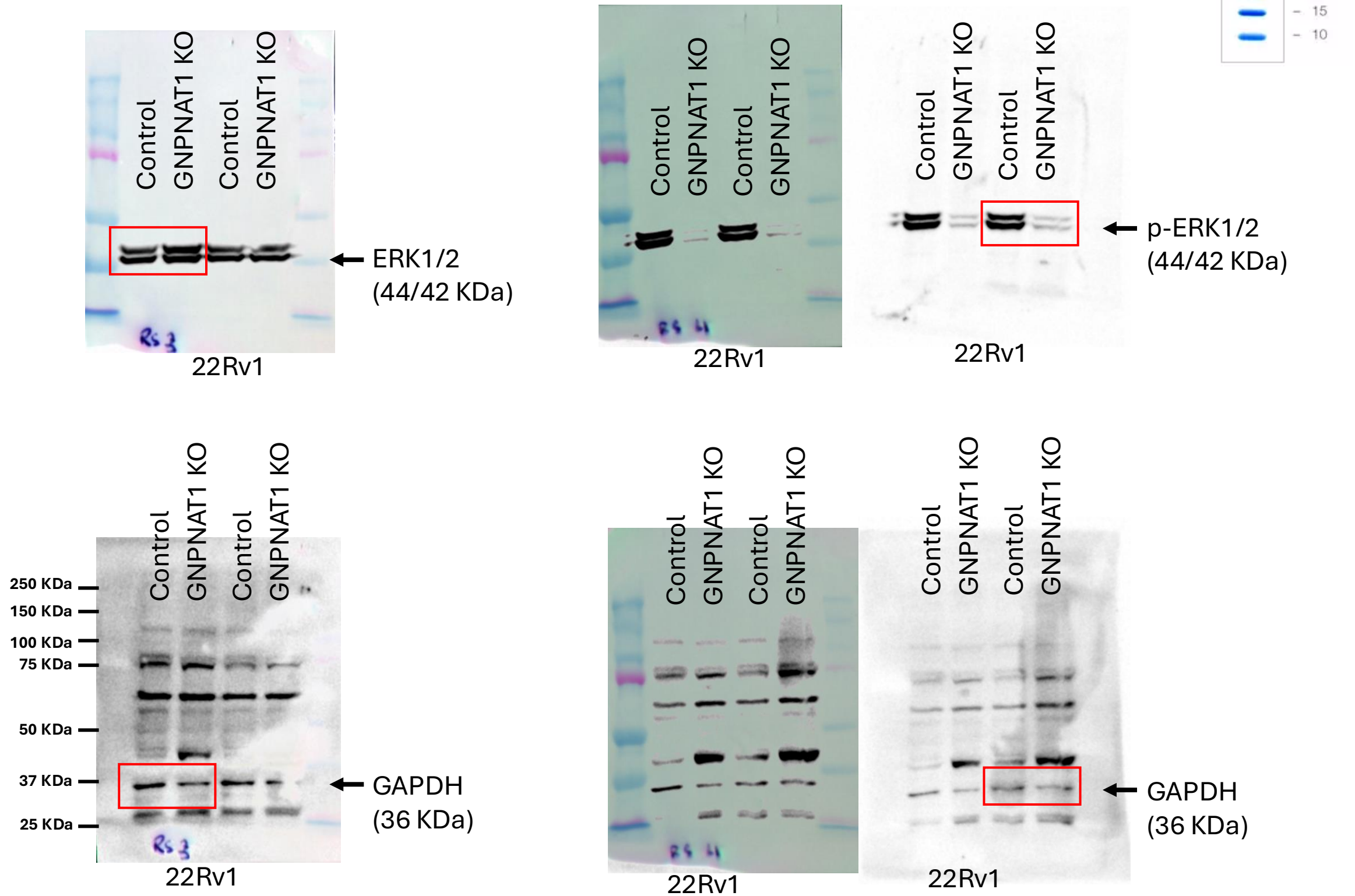

# Figure 6A.

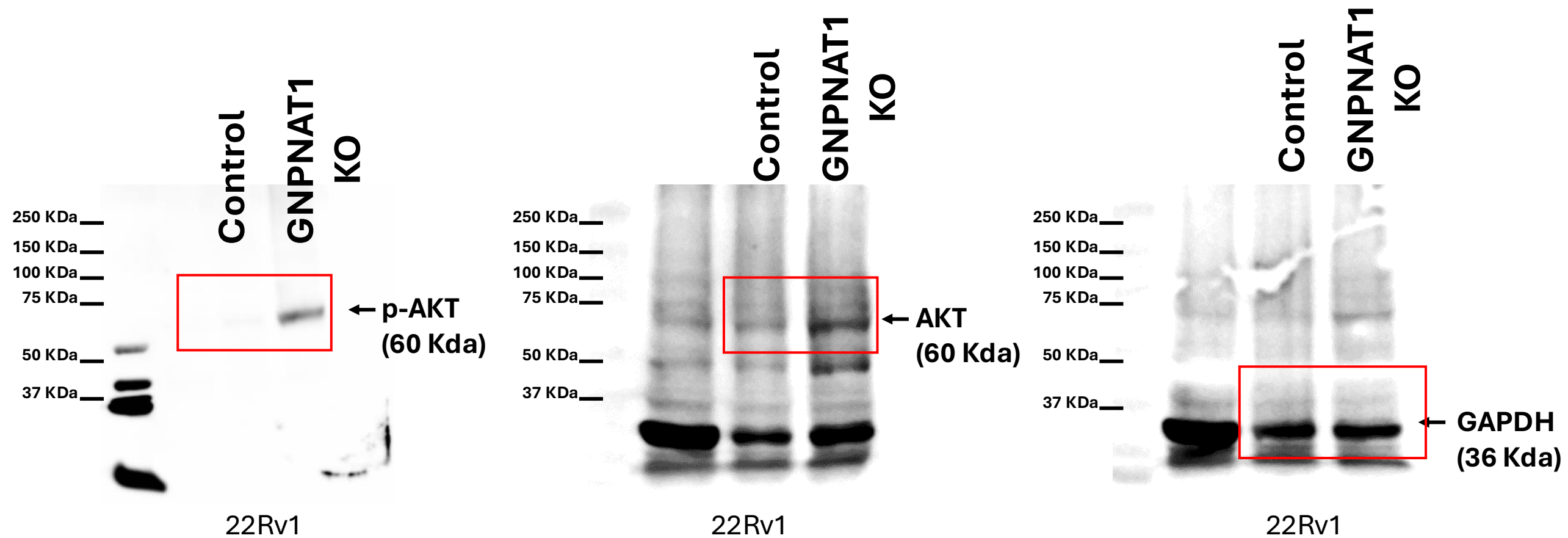

# Supp Figure 1A and B.

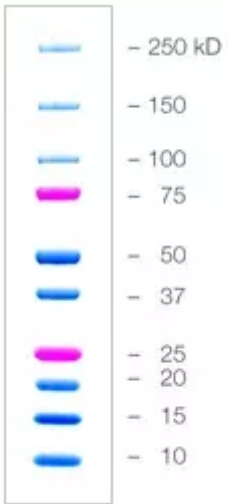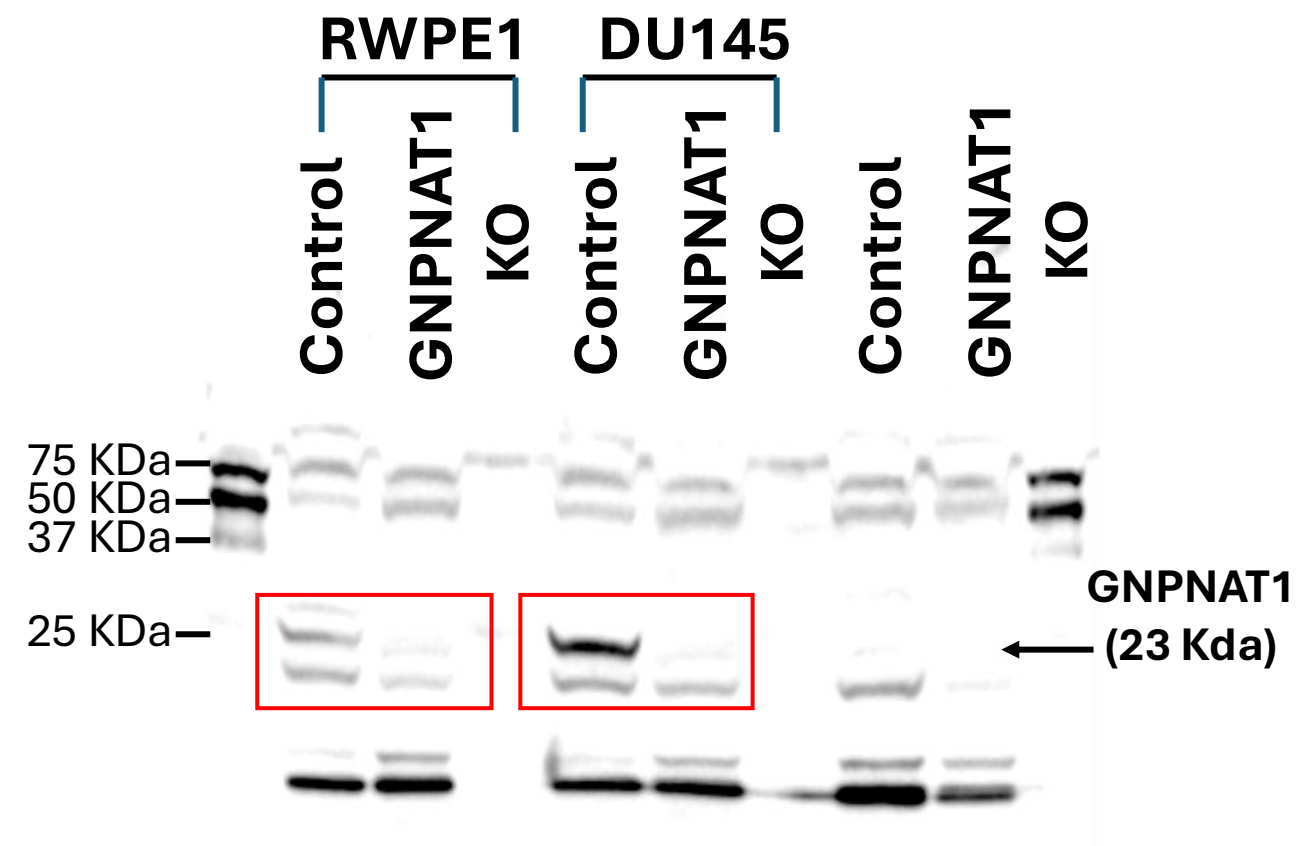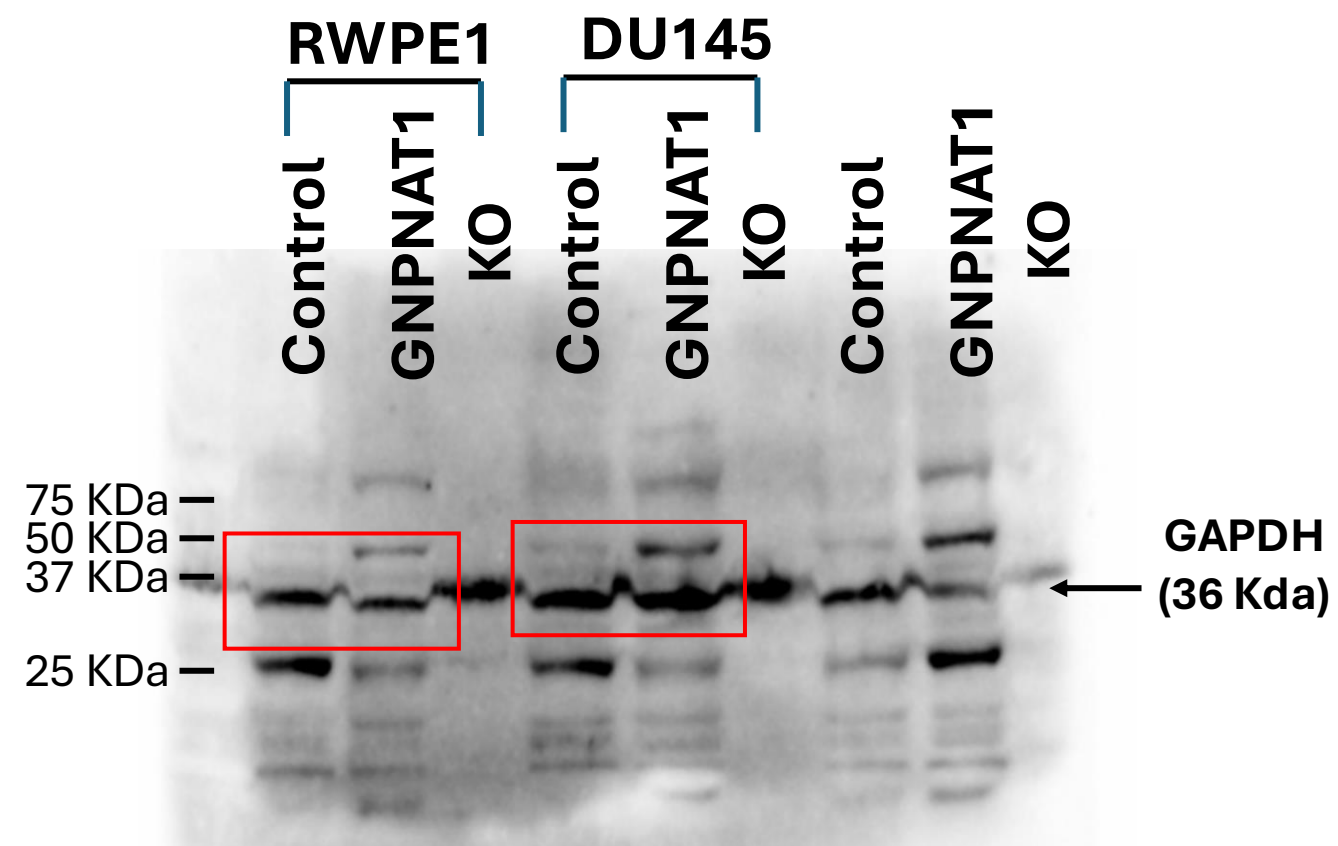

# Supp Figure 7.

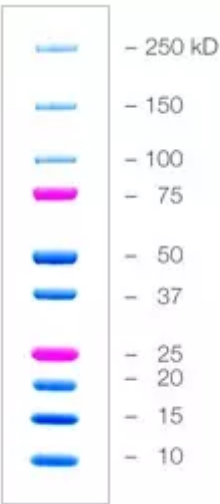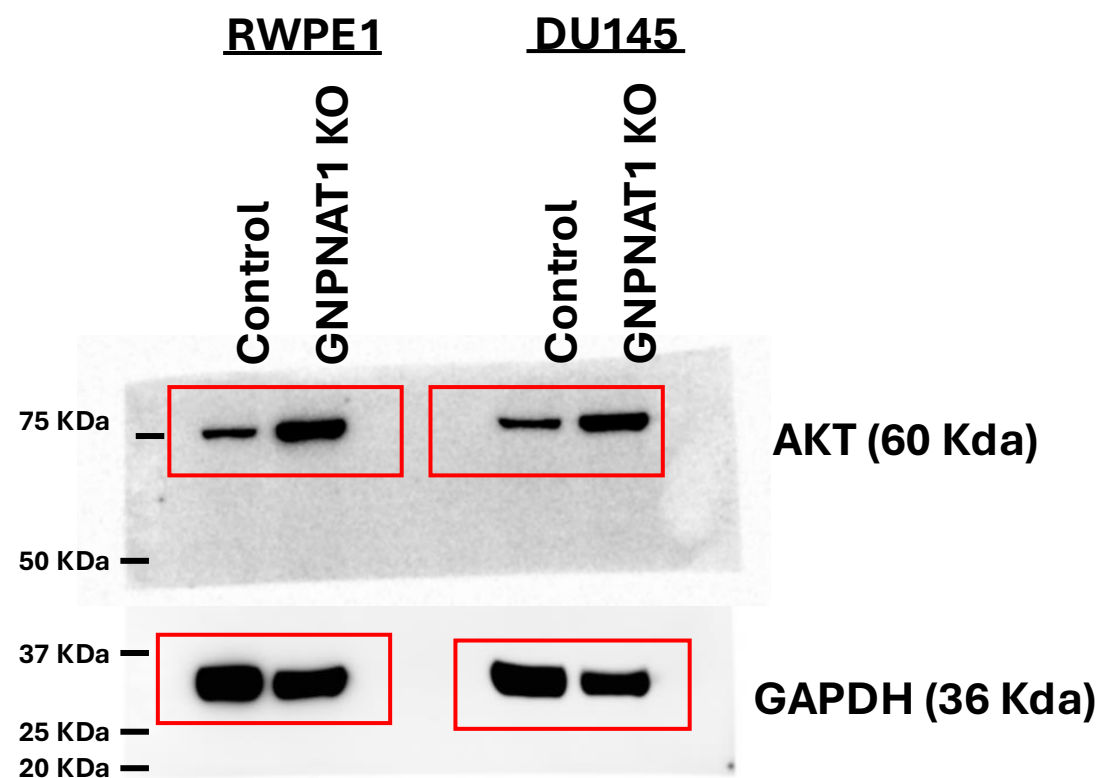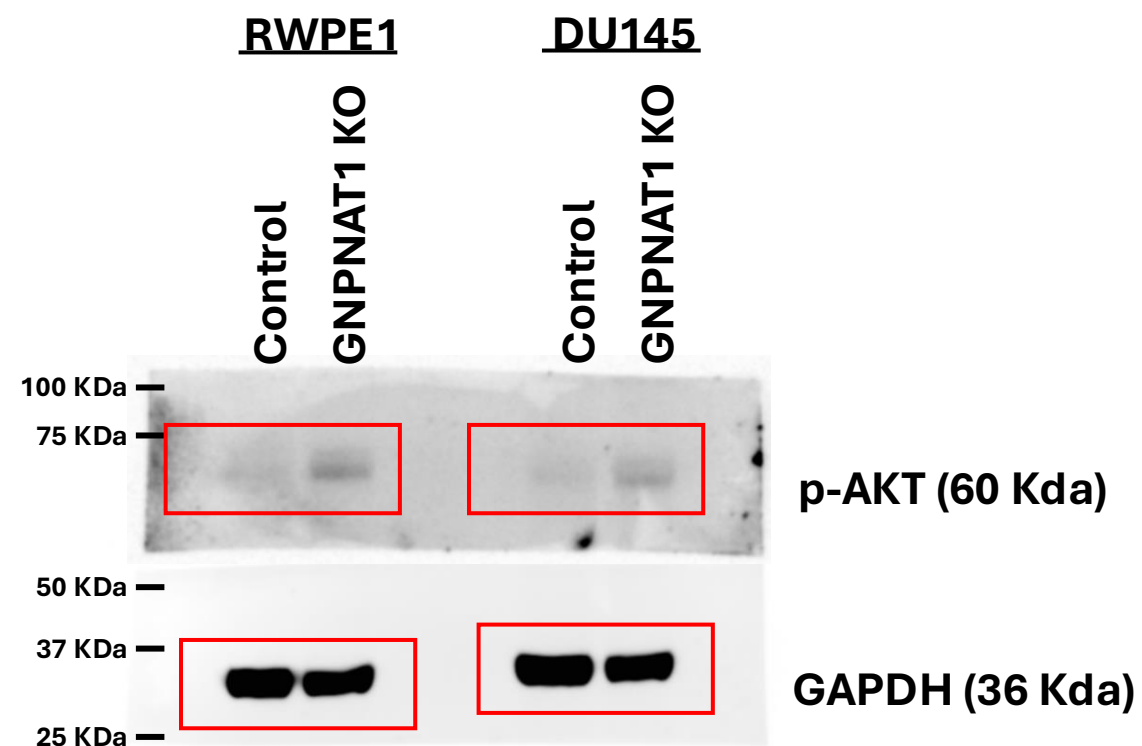

Supplement: Supplementary file 2 — Supplementary Material 2. [file 12964_2026_2756_MOESM2_ESM.pdf]
